# N-phenyl-1-naphthylamine (PNA) Accumulates in Snapping Turtle (*Chelydra serpentina)* Liver Activating the Detoxification Pathway

**DOI:** 10.1007/s00128-020-03043-0

**Published:** 2020-11-19

**Authors:** Tash-Lynn L. Colson, Shane R. de Solla, Vimal K. Balakrishnan, John Toito, Valerie S. Langlois

**Affiliations:** 1 School of Environmental Studies, Queen’s University, Kingston, ON Canada; 2 Ecotoxicology and Wildlife Health Division, Environment and Climate Change Canada, Burlington, ON Canada; 3 Aquatic Contaminants and Research Division, Environment and Climate Change Canada, Burlington, ON Canada; 4 Centre Eau Terre Environnement, Institut national de la recherche scientifique (INRS), 490 de la Couronne, Quebec, QC G1K 9A9 Canada

**Keywords:** Turtle, Toxicity, N-phenyl-1-naphthylamine, Metabolism, Cytochrome P450

## Abstract

Substituted phenylamine antioxidants (SPAs) are used in Canadian industrial processes. SPAs, specifically N-phenyl-1-naphthylamine (PNA), have received very little attention despite their current use in Canada and their expected aquatic and environmental releases. There is a research gap regarding the effects of PNA in wildlife; therefore, *Chelydra serpentina* (common snapping turtle) was studied due to its importance as an environmental indicator species. A chronic experiment was performed using PNA spiked food (0 to 3446 ng/g) to determine its toxicity to juvenile *C. serpentina*. A significant increase in *cyp1a* mRNA level was observed in the liver of turtles exposed to 3446 ng/g PNA, suggesting that phase I detoxification is activated in the exposed animals. Additionally, a significant decrease in *cyp2b* transcript level was observed at the two lowest PNA doses, likely indicating another metabolic alteration for PNA. This study helped determine the molecular effects associated with a PNA exposure in reptiles.

Substituted phenylamine antioxidants (SPAs) have been used in a variety of lubricants, dyes, dispersants, and adhesives to protect the product from oxidation. One SPA, N-phenyl-naphthylamine (PNA), is mainly used as an antioxidant in rubber manufacturing and lubricating oils (Wang et al. 1984). PNA is composed of a naphthalene base with a phenyl group connected by an amine group and is considered lipophilic, as its log K_ow_ is 4.2, and thus it is expected to be slightly bioaccumulative with a bioconcentration factor (BCF) between 50 and 500 (Ozeki and Tejima 1979). Using a fugacity modeling approach, McKay (1991) observed that once PNA is released into the environment, its distribution is predicted to be the following: 36.3% in soil, 33.9% in sediment, 28.9% in water, and 0.016% in air, suspended sediment, and biota. PNA has been measured in sediment and wastewater at concentrations up to 5 and 7 µg/g, respectively (Jungclaus et al. 1978; Lopez-Avila and Hites 1980). More recently, PNA has been measured in biosolids from a wastewater treatment Plant (Hamilton, ON, Canada) at a concentration of 65 ng/g (Balakrishnan et al. 2016); however, environmental concentration data are limited and outdated. As PNA is currently used in industrial processes in open systems and is potentially bioaccumulative and persistent in the environment, it can be considered as a contaminant of emerging concern. PNA was evaluated as part of the Government of Canada’s Chemicals Management Plan, whose purpose is to determine the hazard and risk of chemicals currently used in commerce in Canada. PNA was evaluated as little is known about the environmental exposure, environmental fate, or toxicity of PNA, thus this project was to determine the toxicity to wildlife.

Despite large knowledge gaps of the toxicological significance of PNA, few studies have assessed the effects of this chemical in vertebrates. The majority of the studies on the toxicity of PNA used mammalian models, and the few data that exist on aquatic vertebrates were acute exposures. The EC_50_ for cell proliferation was determined to be 2 mg/L for *Tetrahymena pyriformis* (ciliate) exposed to PNA for 48 h (Epstein et al. 1967). The LC_50_ was in the range of 0.44–0.74 mg/L for *Oncorhynchus mykiss* (rainbow trout) and 0.57–0.82 mg/L for *Lepomis macrochirus* (bluegill). More recently, Prosser et al. (2017) determined that the LC_50_ in *Pimephales promelas* (fathead minnow) was 74 µg/L and the EC_50_ for deformities was 95 µg/L. The LC_50_ and LC_100_ of PNA for *Xenopus laevis* (African clawed frog) were reported to be 2.3 mg/L and 5 mg/L in *Lithobates pipiens* (Northern leopard frog) (Greenhouse 1976, 1977). Furthermore, development of *L. pipiens* exposed to 20 and 200 mg/L PNA was halted at Shumway stage 20, in which death resulted afterwards in 100% of animals (Greenhouse 1976; Shumway 1940). Eye malformations and stunted growth were induced in larval *X. laevis* exposed to > 5.2 mg/L PNA, whereas death occurred when treated with concentrations at, or above 6 mg/L (Greenhouse 1976). All these studies are based upon aqueous exposures, but since PNA is potentially bioaccumulative, dietary exposures may be important. Altogether, these studies suggest that at high exposures of PNA may be a hazard to wildlife, but there is a lack of data on the molecular mechanism of PNA toxicity, most specifically in reptiles.

The goal of this study was to determine if a chronic exposure of PNA in diet to juvenile *Chelydra serpentina* (snapping turtle) would lead to bioaccumulation and alteration of normal physiological functions, such as detoxification, development, and reproduction. It was hypothesized that PNA will be slightly accumulative to turtles and would alter the expression of a subset of genes related to detoxification and endocrine pathways.

## Methods and Materials

Turtle eggs were collected in June 2014, west of Long Point Provincial Park (ON, CA), a site with few local or known sources of contamination. Eggs were incubated at the Canada Centre for Inland Waters (CCIW at Environment and Climate Change Canada (ECCC), Burlington, ON, CA) until hatched. Collection and housing were performed as described in Colson et al. (2021). The animal care protocol was approved by the Animal Care Committee of Queen’s University (Kingston, ON, CA) and followed the guidelines of the Canadian Council of Animal Care.

Trout chow pellets (Martin PROFISHENT™) were treated with PNA (98%; CAS 90-30-2; TCI Chemicals, Portland, OR, USA) using a rotary evaporator (Buchi Vacobox B-177; Taylor Scientific St. Louis, MO, USA) at CCIW (ECCC). A stock solution of 0.1 mg/L PNA was made by dissolving PNA into acetone (99.7% pure, distilled in glass; Caledon Laboratories Ltd., Georgetown, ON). Trout chow was dosed in two batches and placed into the bottom flask with the appropriate volume of stock solution and topped with acetone such that the total volume of the solution was 100 mL. The control food was treated the same way; however, only 100 mL of unadulterated acetone was added to the flask in place of the PNA solution. After mixing the contaminated pellets they were under a fumehood for 30 min, while being incubated in a waterbath at approximately 30 ºC. The pressure of the rotary evaporator was initially set at 556 mbar, and ΔH was set to 1.0, although the pressure was increased near the end of the evaporation. The food mixture was run for about 1 h, occasionally shaken by hand during this time. The treated trout chow was placed on aluminum foil and let sit in a fume hood for 24 h, stored in plastic containers, and kept frozen at − 20 ºC.

Turtle hatchlings (n = 70) were housed in 2.2-L plastic containers with roughly 250 mL of water to allow turtles to submerge, while still allowing easy access for the turtles to breathe. Turtles (n = 14 per treatment) were chronically-exposed to a range of nominal PNA concentrations (0, 0.01, 0.1, 1.0, and 10 µg/g) for 81 days. Each hatchling was fed 5 pellets twice a week for the duration of the experiment.

Turtle hatchlings were sacrificed on day 81 of the exposure by decapitation (Colson et al. 2021). Brain, liver, and GMC (gonad-mesonephros complex) were collected, weighed, and immediately placed on dry ice and stored at − 80 °C until further use. In addition, the whole body was weighed and carapace length was measured for morphometric analyses. Somatic indices were calculated for each collected tissue. Livers were further tested to measure PNA concentrations and mRNA levels.

The extraction and measurement of PNA concentration in turtle liver were performed by liquid chromatography-tandem mass spectrometry (LC-MS/MS; (Balakrishnan et al. 2016). Tissue samples (0.1 g dry weight) were spiked using a SPA solution in MeOH and then evaporated and extracted in 10 mL acetonitrile using ultrasound assisted extraction. Lipids were removed by gel permeation chromatography in columns packed with 30 cm of BioBeads (200–400 mesh; BioRad) that were prepared in 50:50 DCM:Hexane (v/v). Extracts were filtered through Allihn funnels through a 10 cm bed of Celite 545 (Fisher Scientific) on a 1.2 µm Whatman GFC filter (VWR Scientific). Nitrogen was used to dry the filtrate to 1 mL. The extracts were then eluted using 50:50 DCM:Hexane (v/v) in a packed GPC column. Nitrogen was again used to evaporate the DCM:hexane eluate to dryness, after which it was reconstituted in 1 mL MeOH. Samples were analyzed using a XEVO tandem LC triple quadrupole mass spectrometer (Waters, Milford USA) equipped with a Z-Spray electrospray ionization source and operated in the positive-ion mode. MassLynx software (v. 4.1) was utilized for both data acquisition and processing. Multiple reaction monitoring and selected ion reaction modes were used. Aliquots were injected into an UPLC system (Waters, Milford, MA) with a 2.6 µm-pore size Kinetex C18 column (2.1 mm × 100 mm; Phenomenex, USA). All PNA concentrations were normalized against the ^2^H-labeled 1,4-benzene-d_4_-diamine internal standard (internal standard quantification). Total RNA was extracted using TRIzol solution followed by a lithium chloride treatment. DNA contamination was removed by performing DNase I treatment following the manufacturer’s protocol (Promega RQ1 RNase-Free DNase kit; Fisher Scientific, Ottawa, ON, CA). Random primers were used to convert RNA to 1 µg cDNA using and following Promega GoScript™ Reverse Transcription System Kit protocol (Madison, WI, USA). The thermocycle program included an annealing temperature of 25 ºC for 5 min, extending temperature of 42 ºC for 60 min, and 15 min at 70 ºC to inactivate the reverse transcriptase. Samples were kept at − 20 ºC until further use. Eight detoxification-related genes (*i.e., ahr, arnt, cyp1a*, *cyp2b5*, *cat*, *gpx1*, *sod1*, and *hsp70*) were analyzed. Additionally, a subset of thyroid hormone-related genes (*dio2*, *dio3*, *thrα*, and *thrβ*) and sex steroid-related genes (*ar* and *esr1*) were analyzed to determine potential for endocrine disruption. Primers were either obtained from Colson et al. (2021) or Rhen et al. (2007) (Table 1).

**Table 1:** Primer design and conditions for genes involved in detoxification, thyroid hormone, and sex steroid pathways in *C. serpentina*

Function	Gene	Primer direction	Sequence (5′–3′)	Annealing temp (ºC)	Amplicon size (bp)	Primer conc. (µM)	Reference
Normalizing the assay	*odc*	F	GGAGCTACCCTCAAAACTAGC	60	98	0.30	Colson et al. (2021)
		R	GTACAGCCACTTCCAACATGG			0.30	
Detoxification pathway/Oxidative stress	*ahr*	F	GCAACACAGAAACCTCTTACAG	58	101	0.25	
		R	ATACAACACAGCCTCACCAG			0.25	
	*arnt*	F	TCGGATGTTCCCTCTTTGGGT	58	110	0.25	
		R	TCAAGCCCTGGTCGTCTCTT			0.25	
	*cat*	F	CTTGTAGGCAACAACACTCCC	60	103	0.35	
		R	AGATTCAGGACGAAGGCTCC			0.35	
	*cyp1a*	F	ACACAGGCTTCTTAGTCCCTT	58	110	0.35	
		R	TCAGACAGAAGACAGCAGAGG			0.35	
	*cyp2b5*	F	GTGAAGGAAGCCCTGGTGG	60	112	0.35	
		R	CACGTCTCCCCGTTGCTG			0.35	
	*hsp70*	F	TGTTGAAGGAAGGACATCTACCC	62	185	0.35	
		R	CCCTCCAACAATCCCAGCTT			0.35	
	*gpx1*	F	CCTAGGAGAACGCTACCAATG	58	140	0.35	
		R	CAGGAAAGTGAAGAGTGGGTG			0.35	
	*sod1*	F	CTGAAGGAAAACATGGCTTCC	62	118	0.20	
		R	CTCTTTATCCTGTGGTCCACC			0.20	
Thyroid hormone axis	*dio2*	F	GGATGCCTACAAACAGGTCAA	58	115	0.35	
		R	CTTGGTTCCATATTTCCCGCC			0.35	
	*dio3*	F	CTGAAGGAAAACATGGCTTCC	58	91	0.30	
		R	CCATGGTGTCCACTGCCAG			0.30	
	*thra*	F	GCAAGGAGGAGATGATCAAGAC	58	104	0.35	
		R	TTCCGCTTCTGTTTCCA			0.35	
	*thrb*	F	CCAGTGCCAGGAATGTCGCTT	60	123	0.35	
		R	CGTCTCTTCTCTCGGTTTTCT			0.35	
Sex steroid axis	*ar*	F	TGGGATGGAGATCTTTCACCAA	58	52	0.35	Rhen et al. (2007)
		R	GGAGCAAAGTAAAGCATCCGG			0.35	
	*esr1*	F	AACCAGTGCACCATCGACAAG	58	103	0.20	
		R	AATCTTTTCGGATCCCACCTT			0.30	

F: forward primer, R: reverse primer. *odc*: ornithine decarboxylase, *rpl8*: ribosomal protein L8, *ahr*: aryl hydrocarbon receptor, *arnt*: aryl hydrocarbon receptor nuclear translocator, *cat*: catalase, *cyp1a*: cytochrome P450 1a, *cyp2b5*: cytochrome P450 2b5, *hsp70*: heat shock protein 70 kDa, *gpx1*: glutathione peroxidase 1, *sod1*: superoxide dismutase 1, *dio1*: iodothyronine deiodinase 1, *dio2*: iodothyronine deiodinase 1, *thra*: thyroid hormone receptor alpha, *thrb*: thyroid hormone receptor beta, *ar*: androgen receptor, *esr1*: estrogen receptor 1

Gene expression analysis was measured on an Agilent Mx3005P Real-Time PCR (qPCR; Agilent Technologies, Inc., Santa Clara, CA, USA) using the Promega GoTaq Bryt® Green qPCR Master Mix (2X; Fisher Scientific). For each qPCR assay, a negative template control and a negative reverse transcriptase control were included to ensure no contamination. A standard curve was prepared through serial dilution (1:4) starting at 50 ng. All samples, controls, and the standard curves were run in duplicate. Efficiencies ranged between 83–122%, and coefficients of determination (R^2^) were above 0.983. Gene expression was normalized to the quantified relative expression of *odc*. Gene expression changes were reported as fold changes relative to the controls.

Statistical analysis of gene expression was done using Prism GraphPad Prism 6 (GraphPad Software Inc, San Diego, CA, USA). Observations that were outside 1.5× interquartile range (IQR) were removed as outliers prior to analysis and data were transformed (log_10_ or square root) if not normally distributed. Comparisons of gene expression analysis among treatments were performed using a one-way ANOVA followed by Tukey’s HSD test. Treatments were considered significantly different if *p*-values were equal to, or below 0.05.

## Results and Discussion

**Fig. 1:**
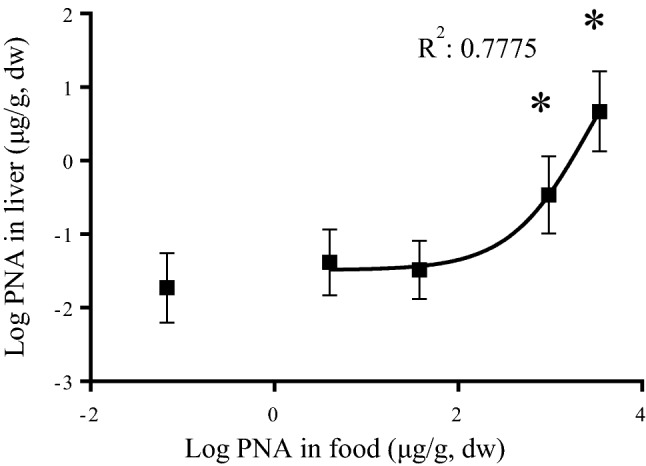
PNA accumulation in the *C. serpentina* liver exposed to varying concentrations (0–3,446 µg/g PNA, dw) after 81 days. Data is presented as mean (n = 7–9) ± SD. A significant sigmoidal increase was noted p < 0.05 and denoted by an asterisk (*) after a one-way ANOVA

PNA concentrations were measured in food pellets to determine the actual dose given to *C. serpentina*. Mean concentrations in liver for each treatment were 0.02, 0.54, 0.05, 0.55, and 7.62 µg/g, dry weight when exposed to pellets of 0, 4, 38, 964, and 3,446 µg/g, dry weight PNA, respectively (Fig. 1). A significant increase was observed in PNA concentrations in liver (R^2^: 0.78; Fig. 1). Livers from the two highest treatments accumulated significantly more PNA than the control (0.55 and 7.62 µg/g compared to 0.02 µg/g, respectively). In addition, the liver accumulation factors (liver concentration/concentration in food) calculated for the two highest treatments were 0.006 and 0.0023 in liver, respectively.

A range of morphometric measurements were taken at experiment completion to assess if PNA altered growth in juvenile *C. serpentina*. Body mass ranged between 9.2 and 19.2 g, while carapace length ranged from 3.2 to 4.1 cm across treatments, with averages of 13.6 g and 3.7 cm, respectively. The gonadosomatic index (GSI) ranged from 0.07 to 0.56 with an average of 0.28. The mean GSI was significantly larger in the highest PNA treatment (3446 µg/g PNA) when compared to that of controls which suggests that PNA exposure can increase gonadal mass. Mahboob and Sheri (2002) have determined that the GSI is a good indicator of gonadal enzymatic activity. However, given the lack of research on PNA or related SPAs, no other studies have reported any change in GSI or gonad mass so far. Therefore, the present study suggests that PNA can increase gonadal growth; however, due to the lack of expression changes in *ar* or *esr1* mRNA levels, the observed gonadal growth was unlikely mediated through differential androgen- or estrogen-receptor mRNA expression. Further research would be required to investigate this increase in gonadal mass. No other significant differences were found for any other morphometric endpoints.

**Fig. 2:**
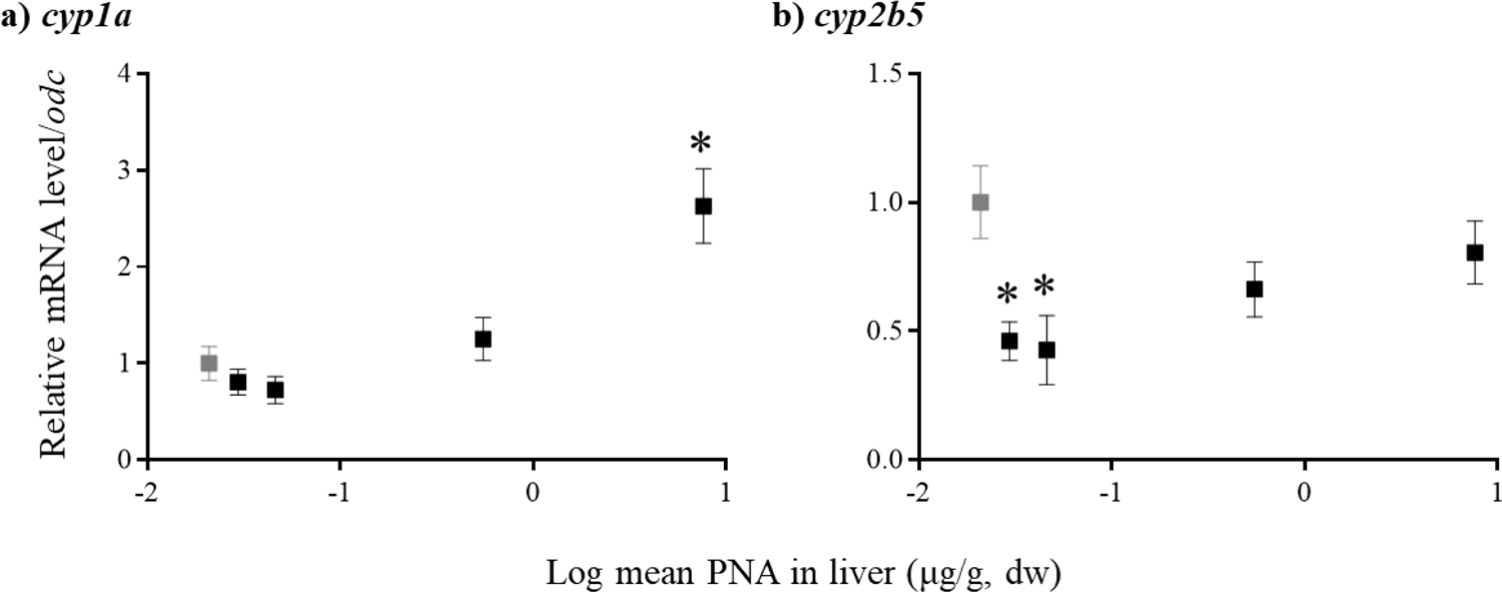
Cytochrome P450 gene expression in *C. serpentina* liver after exposure to 0 (gray box) and 4–3446 (black boxes) µg/g PNA. Data are presented as mean fold change + SEM. Significance (p < 0.05) compared to control is depicted by an asterisk (*) after a one-way ANOVA and Tukey’s test. *cyp1a* = cytochrome P450 1A; *cyp2b5* = cytochrome P450 2B5

Fourteen genes were targeted to assess detoxification, oxidative stress-, thyroid hormone-, and reproductive-related pathways. A significant 2.7-fold increase (*p* = 0.0003) in *cyp1a* mRNA level was observed after exposure to 3,446 µg/g PNA (Fig. 2a). This may indicate that PNA can be detoxified through phase I metabolism. CYP1A is the enzyme responsible for the addition of hydroxyl groups during xenobiotic metabolism. This increase in *cyp1a* expression in *C. serpentina* suggests that PNA may be metabolized through hydroxylation in the turtle liver. For example, hydroxylated metabolites of PNA were detected in rat microsomes following in vitro exposure (Xuanxian and Wolff 1992). Taken together, the increase in *cyp1a* mRNA level measured in this study and the susceptibility of PNA to be metabolized into hydroxylated metabolites, suggest that PNA is likely metabolized in the liver via the CYP1A pathway.

In contrast, significant 0.5- and 0.4-fold decreases (*p* = 0.0084) were observed in *cyp2b5* transcript level after exposure to 4 and 38 µg/g PNA, respectively (Fig. 2b). No other changes in gene expression were observed. Most interestingly, a U-shaped response was observed for *cyp2b5* transcript level with initial decreases in *cyp2b5* mRNA levels at low doses and then a return to control levels at higher doses, which suggests a hormetic response. Hormesis is a dose-response relationship in which the response resembles a U-shape or an inverted U-shape due to stimulation at low doses but inhibition at high doses (Davis and Svendsgaard 1990). Many underlying mechanisms may be responsible for hormesis, such as an overcompensation to maintain homeostasis at low doses of a toxicant (Calabrese and Baldwin 2001). For example, exposure to dioxin-like compounds can create a U-shape response at low doses, which coincides with the multiple different effects of dioxins, such as cell proliferation, toxicity, and mitosuppression for tumour induction (Andersen and Barton 1998). Further investigation would be needed to validate and explain the inverted U-shaped response measured for *cyp2b5* in *C. serpentina* livers.

Despite the alterations observed for the expression of the two *cyp* genes analyzed, no other changes were noted for any of the phase II detoxification- or oxidative stress- related genes investigated. This can be partly explained by the turtle’s high tolerance to oxidative stress. *C. serpentina* are known to have high basal antioxidant defenses (i.e., CAT, SOD, and GST) (Hermes-Lima and Zenteno-Savin 2002). This high level of antioxidants allows turtles to resist long periods of stress, such as hibernation, in which they undergo anoxia (Storey 1996), in which reactive oxygen species are generated during periods of high oxygen tension during reoxygenation (Krivoruchko and Storey 2010).

This is the first study to determine if PNA is toxic to reptiles, and more specifically, in turtles. Overall, our data have shown that PNA accumulates, albeit slightly, in the turtle liver and suggests that it can be likely metabolized by P450 enzymes. Further investigation is needed to understand the exact detoxification mechanisms of PNA in juvenile turtles. Understanding how emerging contaminants, such as PNA, can affect wildlife is imperative to assist environmental risk assessment to prevent negative health consequences to wildlife populations.
